# Effects of endophytic fungi diversity in different coniferous species on the colonization of *Sirex noctilio* (Hymenoptera: Siricidae)

**DOI:** 10.1038/s41598-019-41419-3

**Published:** 2019-03-25

**Authors:** Lixiang Wang, Lili Ren, Chunchun Li, Chenglong Gao, Xiaobo Liu, Ming Wang, Youqing Luo

**Affiliations:** 1 Beijing Key Laboratory for Forest Pest Control, Beijing Forestry University, Beijing, 100083 China; 2 Sino-France Joint Laboratory for Invasive Forest Pest in Eurasia, Beijing, 100083 China; 3 Shangluo Agricultural Mechanical Technology Extension Station, Shangluo, 726000 China

## Abstract

Diversity of endophyte communities of the host tree affects the oviposition behavior of *Sirex noctilio* and the growth of its symbiotic fungus *Amylostereum areolatum*. In this study, we evaluated the structure and distribution of endophyte communities in the host tree (*Pinus sylvestris* var. *mongolica*) of *S. noctilio* and eight potential host tree species in China. Overall, 1626 fungal strains were identified by using internal transcribed spacer sequencing and morphological features. Each tree species harbored a fungal endophyte community with a unique structure, with the genus *Trichoderma* common to different communities. The isolation and colonization rate of endophytes from *Pinus tabulaeformis*, followed by *P. sylvestris* var. *mongolica*, were lower than those of other species. The proportion of endophytic fungi that strongly inhibited *S. noctilio* and symbiotic fungus growth was significantly lower in *P. tabulaeformis*, *P. sylvestris* var. *mongolica* and *P. yunnanensis*. Further, the diversity of the endophyte communities appeared to be predominantly influenced by tree species and the region, and, to a lesser extent, by the trunk height. Collectively, the data indicated that *P. tabulaeformis* might be at a higher risk of invasion and colonization by *S. noctilio* than other trees.

## Introduction

Endophytic fungi are microorganisms that live in plant tissues without causing apparent harm to the host^1,2^. Therefore, harboring of the endophytic fungi by plants is asymptomatic^3^. These fungi are part of the plant microbiome and are ubiquitously found across plant species and ecosystems^4^. In addition, endophytes appear to be closely associated with different parts of the host plant, characteristic of the locality where the plant grows^5^, and distribution of the host plant^6^. Among the xylophyta, endophytic fungi can produce a range of metabolites, some of which exert inhibitory effects on pests and pathogens. Endophytic fungi are also able to promote tree growth, and contribute to tree stress acclimation and yield^7^. Wang^8^ demonstrated that endophytic fungi have potential as bio-control agents because they might produce antifungal substances that are capable of inhibiting the growth and spore germination of microbial pathogens. For this reason, endophytic fungi are considered as a promising natural resource of future bio-control agents for forestry management^9^.

The European woodwasp, *Sirex noctilio* Fabricius (Hymenoptera: Siricidae), is a global invasive pest distributed across the six continents. It tends to attack pine species, mainly including *Pinus pinaster*, *P. radiata*, *P. elliottii*, *P. sylvestris* and *P. taeda*, and occasionally was found to attack *Picea* spp., *Abies* spp. and *Larix* spp^10–13^, which results in considerable economic and ecological damage^14,15^. Female woodwasp damages trees by depositing a phytotoxic mucus and an obligate symbiotic fungus, *Amylostereum areolatum* (Fr.) Boidin (Basidiomycotina: Corticiaceae), in the trees during oviposition. The growth and development of *S. noctilio* larvae is correlated with the growth of the symbiotic fungus. The larvae feed exclusively on the fungus until the third instar and then feed on fungus-colonized wood. August 2013 marks the first instance when *S. noctilio* was found to damage *Pinus sylvestris* var. *mongolica*, the primary afforestation tree species in the northeastern and northwestern regions of China. To date, *P. sylvestris* var. *mongolica* plantations spanning 22 cities in northeastern China have been considered endangered by *S. noctilio*, and enormous economic and ecological losses were noted. In addition, *S. noctilio* was intercepted in imported wooden packing from Slovenia for the first time at the Huizhou port of Guangdong (southern China) in August 2017^16^. In Australia and New Zealand, *S. noctilio* mainly attacked exotic pine plantations^17^. Research suggests that native and exotic *Pinus* stands (*Pinus taeda* and *Pinus elliottii*) in China may have a high risk of incursion by *S. noctilio*, assuming that it is not native to the area^17,18^.

Endophytic fungal colonization of the host plant impacts insect abundance. Namely, the numbers of some insects decrease with a consecutive increase of the numbers of endophytic fungi^19^. Resistance of the endophytic fungi to the woodwasp and competition with the symbiotic fungus of the woodwasp likely contribute to keeping the *S. noctilio* population in North America below damaging levels to a greater extent than the natural enemies of the woodwasp^20^. However, *A. areolatum* grows slowly and the ability of occupying the niche is weaker than the other fungi. Fungi including *Leptographium wingfieldii*, *Ophiostoma minus*, *Sphaeropsis sapinea*, and *L. procerum* inhibit the mycelial growth of *A. areolatum*, they could not completely replace *A. areolatum* colonies when plated in direct contact with *A. areolatum*^21–23^. Among these fungi, *L. wingfieldii* and *O. minus* affect the selection of the oviposition sites by the woodwasp^24^. Interestingly, we found that some fungal endophytes (*Trichoderma* sp. and *Phlebiopsis gigantea*) of *P. sylvestris* var. *mongolica* completely killed the mycelia of *Amylostereum areolatum*^25^. Moreover, we observed that volatiles of *Ophiostoma minus* and *Aspergillus niger* had repellent effects on adult female woodwasp^25^. However, some other endophytic fungi also enhance the activity of the invasive insect, resulting in an enhanced insect abundance, greater rates of parasitism, and increased strength of interactions at high trophic levels^26^.

*Pinus sylvestris* var. *mongolica* is the only host of *S. noctilio* in the mixed coniferous forest (*P. sylvestris* var. *mongolica*, *Pinus koraiensis*, *Picea koraiensis* (henceforth was abbreviated as *Pc*. *koraiensis*) and *Larix gmelinii*) in China^27^. Endophytic fungi in woodwasp-infested *P. sylvestris* var. *mongolica* differ from those in uninfested trees that exhibit the same vitality and are located in the same stand as the infected trees^28^. Furthermore, *S. noctilio* mainly destroys the host trunk at a height of 2~4 m. Diversity of endophyte communities of the host tree affects the growth and development of *S. noctilio* larvae^25^. Interestingly, the host selection experiment of *S. noctilio* (laboratory) revealed that the adult female woodwasp preferentially lays eggs on logs of *Pinus tabulaeformis*, *P. sylvestris* var. *mongolica*, and *Pinus massoniana*. The proportion of spawning on *P. tabulaeformis* is significantly higher than that on other coniferous trees (unpublished data, Xiaobo Liu). In addition, the woodwasp completes its entire life cycle in *P. tabulaeformis*, *P. sylvestris* var. *mongolica*, and *Pinus yunnanensis* logs. Furthermore, we found numerous *S. noctilio* larvae infected by fungi that failed to develop, and eventually died in the host tree. However, the relationship between *S. noctilio* host selection, and the distribution and diversity of endophytic fungi in different trees has not yet been elucidated, and, hence, it is difficult to comment on whether *S. noctilio* damages other tree species in China.

In the current study, nine major coniferous species in China were selected, i.e. the host tree of *S. noctilio* and others (*P. tabuliformis*, *Pinus yunnanensis*, *Pinus massoniana*, *P. taeda*, *P. elliottii*, *P. koraiensis*, *Pc. koraiensis*, and *L. gmelinii*). Based on the results of host selection experiments of *S. noctilio*, the aim of the study was to compare the diversity and difference of endophytic fungi in host species and potential host species of *S. noctilio*, and to provide a basis for evaluating the invasion risk of other conifers by *S. noctilio* in China. Further, the study of fungi associated with coniferous trees identified the presence of biocontrol fungus and provided information on how some of these endophytic fungi could be potentially used to control the *S. noctilio* population on important cultivated trees.

## Results

### The occurrence of fungal endophytes in different tree species

**Figure 1:**
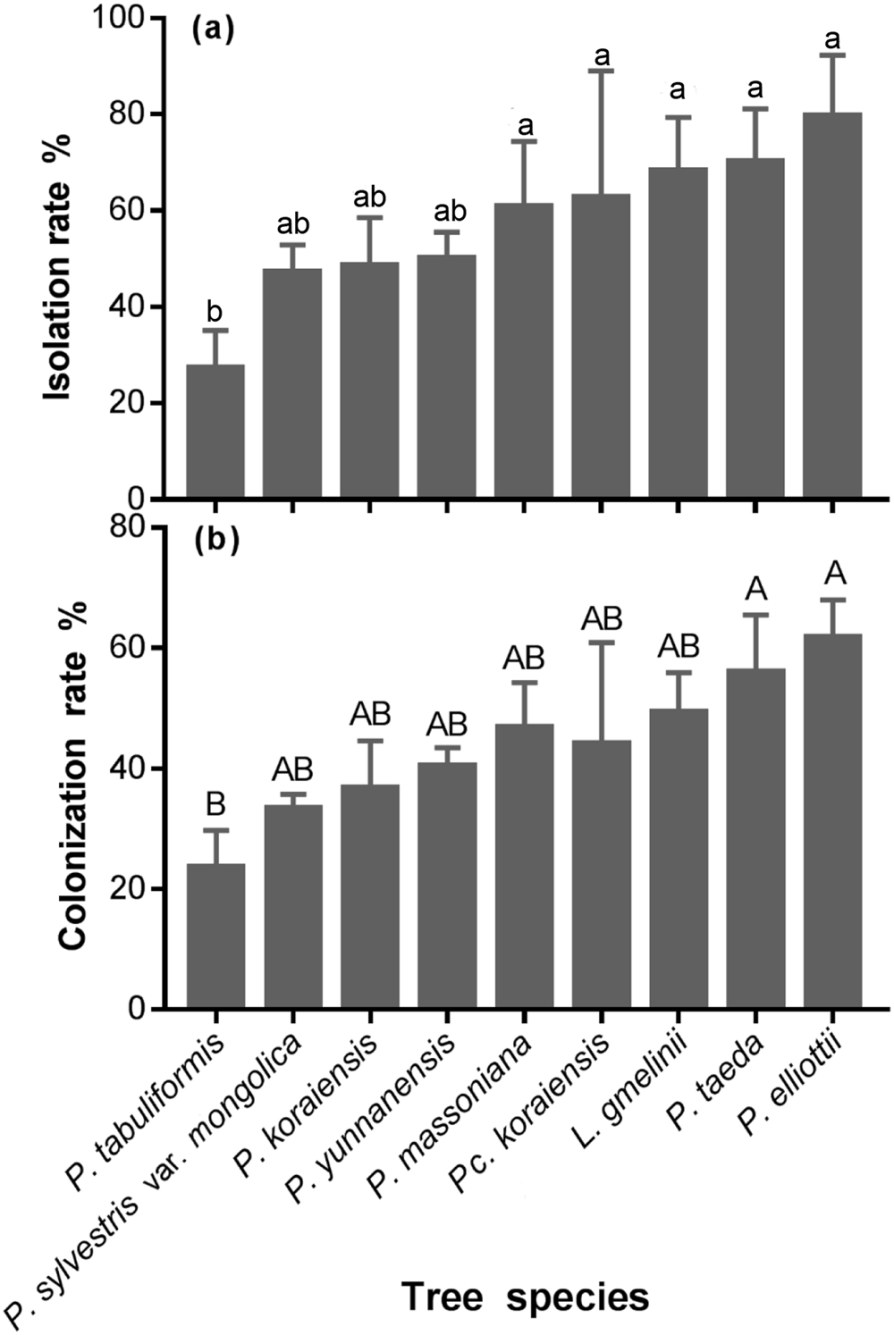
The rates of isolation (**a**) and colonization (**b**) of endophytic fungi from nine tree species. Lowercase letters indicate a significant difference between the isolation rates in different tree species at p < 0.05. Uppercase letters indicate a significant difference between the colonization rates in different tree species at p < 0.01.

Overall, 1626 endophytic fungal strains were isolated from 2025 wooden blocks of nine coniferous tree species. The colonization rate (CR) and isolation rate (IR) of different species were significantly different (CR: F = 3.91, p < 0.01; IR: F = 4.9, p < 0.05) (Fig. 1). In HG, the CR (33.4%) and IR (47.2%) of endophytes of *P. sylvestris* var. *mongolica* were the lowest compared to other three conifers (CR: *χ*^2^ test, p < 0.05; IR: *χ*^2^ test, p < 0.01). Among seven species of pine trees, the CR and IR of endophytes from *P. tabuliformis* were the lowest, and those from *P. elliottii* were the highest. The CR of *P. taeda* (56%) and *P. elliottii* (61.7%) were significantly higher than that of *P. tabuliformis* (23.6%, *χ*^2^ test, p < 0.01) (Fig. 1). The IR of *P. elliottii* (79.7%), *P. taeda* (70.1%), *P. massoniana* (60.8%), and that of *P. tabuliformis* were significantly different (27.2%, *χ*^2^ test, p < 0.01). There were no significant differences in the CR or IR between *P. sylvestris* var. *mongolica* and the remaining six species of pine trees.

### The structure of fungal endophyte communities

The isolated 1626 endophytic fungi were assigned to 61 species and 34 genera (Table 1). Fifty-one species (83.6%) were identified based on ITS sequencing and the other ten species (16.4%) were identified based on morphological features. In HG, the endophyte richness of *Pc. koraiensis* (fifteen genera) was the highest, and that of *P. koraiensis* was the lowest with seven genera. The prevalence of *Fusarium* (2.9%) and *Trichoderma* (7.7%) of *P. sylvestris* var. *mongolica* was considerably lower than the other three tree species, while *Aspergillus* was more prevalent in *P. sylvestris* var. *mongolica* compared to the other three species. The endophyte richness was similar in JDZ (*P. taeda*, five genera; *P. elliottii*, six genera; and *P. massoniana*, six genera). The most prevalent were *Penicillium* and *Trichoderma*, which were detected in all tree species in JDZ. The endophytic fungi of *P. tabuliformis* in TL were identified into eight genera, and *P. yunnanensis* in DL were identified into ten genera (Table 1). The particularly common genera were *Trichoderma*, *Penicillium*, *Aspergillus*, and *Fusarium*. *Trichoderma* isolated from nine tree species. However, 16 genera only were shared from different tree species, accounting for 47% of all the genera.

**Table 1:** Genera of endophytic fungi from nine coniferous trees and their isolation rates (%).

Genus	*Pinus tabuliformis*	*Pinus sylvestris var. mongolica*	*Pinus koraiensis*	*Picea koraiensis*	*Larix gmelinii*	*Pinus yunnanensis*	*Pinus massoniana*	*Pinus taeda*	*Pinus elliottii*
*Alternaria*	1.3	7.2	0	2.9	1.6	5.9	0	0	0
*Arthrinium*	0	0	0	0	0	0	1.1	0	0
*Ascocoryne*	0	0	4.5	0	0	0	0	0	0
*Aspergillus*	17.1	9.6	1.1	5.6	3.7	12.3	0	0	30.9
*Capronia*	0.3	0	0	0	0	0	0	0	0
*Chaetomium*	0	9.1	2.7	0	0	1.9	0	0	0
*Coprinopsis*	0.8	0	0	0	0	0.5	0	0	0
*Cylindrobasidium*	0	0	1.1	0	0	0	0	0	0
*Cytospora*	0	0	0	0	6.4	0	0	0	0
*Diaporthe*	0	0	0	1.6	0	0	0	0	0
*Fusarium*	0	2.9	4.3	7.5	15.7	0	0	3.2	3.7
*Gliocladium*	0	0	0	0	0	0	0	4.3	0
*Gliomastix*	0	0.5	0	1.6	0	0	0	0	0
*Lasiodiplodia*	0	0	0	0	0	0	0	14.4	2.9
*mucor*	0	0	0	0	28.2	2.9	4.3	0	0
*Ophiostoma*	0	0	8.3	0	0	0	6.7	0	0
*Paecilomyces*	0	0	0	0	0	1.1	0	0	0
*Paraconiothyrium*	0	0	0	3.7	0	0	0	0	0
*Penicillium*	3.5	3.5	0	5.6	2.7	14.9	12.8	5.9	8.5
*Pestalotiopsis*	0	0	0	0.8	0	0	0	0	0
*Peyronellaea*	0	2.4	0	0	0	0	0	0	0
*Phaeomollisia*	0	0	0	2.6	0	0	0	0	0
*Phoma*	0	0	0	3.5	0	0.3	0	0	0
*Phomopsis*	0	0	0	1.3	0	0	0	0	0
*Pythium*	0.8	0	0	0	0	0	0	0	0
*Schizophyllum*	0.5	0	0	0	0	0	0	0	0
*Seiridium*	0	0	0	0.5	0	0	0	0	0
*Sphaeropsis*	0	4.3	0	9.6	0.5	0	12.8	0	3.2
*Sydowia*	0	0	0	0.3	0.3	0	0	0	0
*Trichoderma*	3.9	7.7	26.7	15.5	9.1	9.1	23.2	42.4	30.5
*Umbelopsis*	0	0	0	0	0	1.3	0	0	0

**Figure 2:**
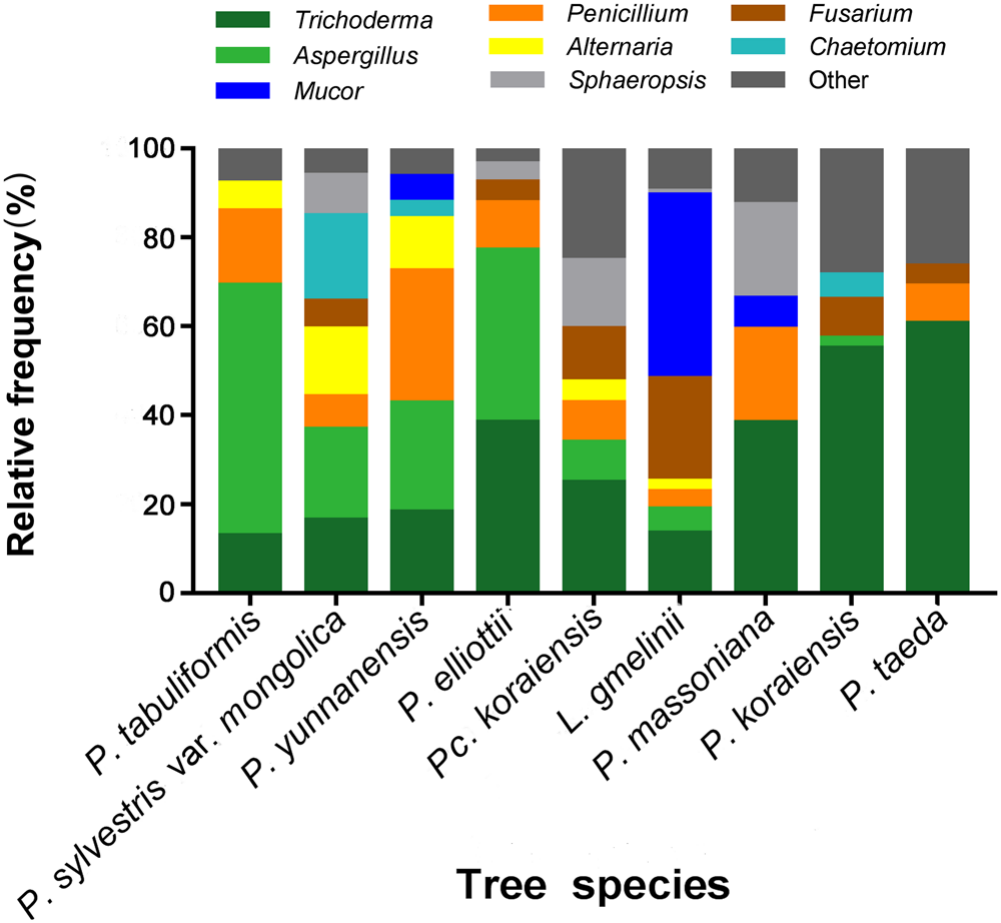
Composition of the most frequently isolated genera from the different tree species. The eight most frequently cultivated genera were selected and the prevalence (%) of each genus was determined per tree species.

The top-eight most prevalent genera accounted for 84% of all the isolates, and between 73% and 96% of the isolates from each tree species (Fig. 2). The proportion of *Trichoderma* was less than 10% in *P. tabuliformis*, *P. sylvestris* var. *mongolica*, *P. yunnanensis* and *L. gmelinii*, and *P. tabulaeformis* was the lowest with 3.9%; The proportion of *Trichoderma* was over 20% in *P. koraiensis*, *P. taeda*, *P. elliottii* and *P. massoniana*, and *P. taeda* was the highest with 42.4% (Table 1; Fig. 2). *Aspergillus* accounted for a high proportion of isolates in *P. tabulaeformis* (17.1%), *P. sylvestris* var. *mongolica* (9.6%), *P. yunnanensis* (12.3%), and *P. elliottii* (30.9%). The proportion of *Mucor* was highest in *L*. *gmelinii* with 28.2%.

### Vertical distribution of the endophytic fungi

**Figure 3:**
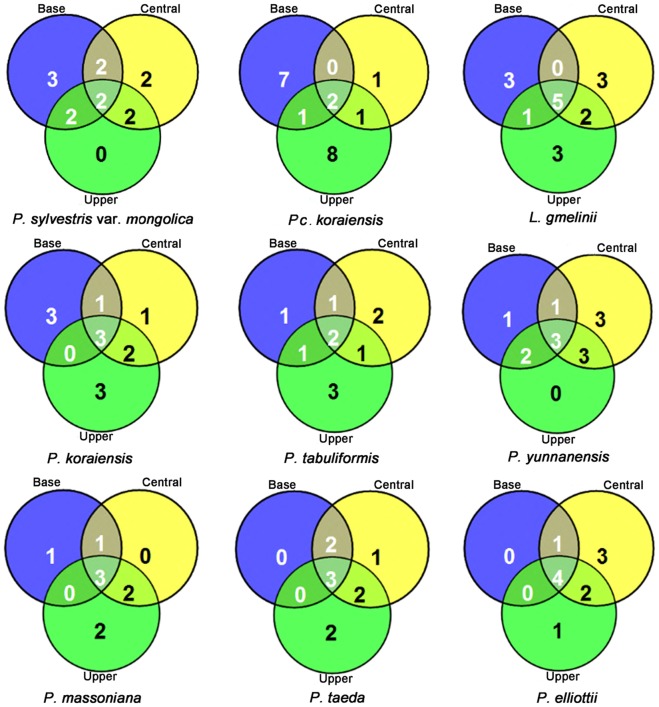
Distribution of cultivated endophytes according to the trunk height (Base, Central, and Upper) in the different tree species. Venn diagrams of the endophytic fungi were produced based on the endophytic fungal presence or absence data. The numbers correspond to number of species of endophytic fungi.

The richness of endophytic fungi isolated from the base trunk segment was higher than that from the central and upper trunk segments in HG trees (Fig. 3). Considering the tree species, the structures of endophyte communities at different trunk heights of *Pc. koraiensis* and *L. gmelinii* were more complex than those from *P. sylvestris* var. *mongolica* and *P. koraiensis*. The richness of endophytic fungi at different trunk heights in trees from TL and DL was more homogeneous. In JDZ trees, the richness of endophytic fungi was lower in the base trunk segment than in the central and upper segments. The most prevalent fungi at different trunk heights were *Fusarium tricinctum* and *T. atroviride* in HG; *Aspergillus versicolor* in TL; *A. niger* and *Penicillium chrysogenum* in DL; and *T. citrinoviride* in JDZ (Fig. 3; Table S1, Supporting Information). The same fungal species has not been found in all the trees in the current study.

### The diversities and similarities of endophytic fungal communities from four conifers invaded by *S. noctilio* in HG

**Figure 4:**
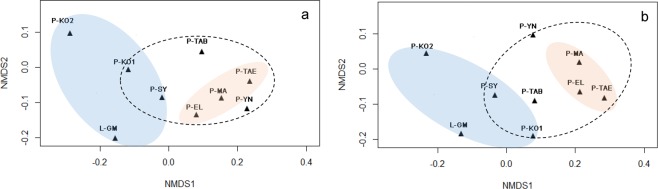
NMDS plots of fungal endophytic communities from nine tree species. NMDS plots based on the Jaccard’s index (**a**) and Bray–Curtis coefficient (**b**) are shown. Blue area: four coniferous trees in HG; yellow area: three trees species in JDZ; dotted area: seven species of pine trees. Abbreviations: P-SY, *P. sylvestris* var. *mongolica*; P-KO1, *P. koraiensis*; P-KO2, *Pc. koraiensis*; L-GM, *L. gmelinii*; P-TAB, *P. tabuliformis*; P-YN, *P. yunnanensis*; P-MA, *P. massoniana*; P-TAE, *P. taeda*; and P-EL, *P. elliottii*.

**Figure 5:**
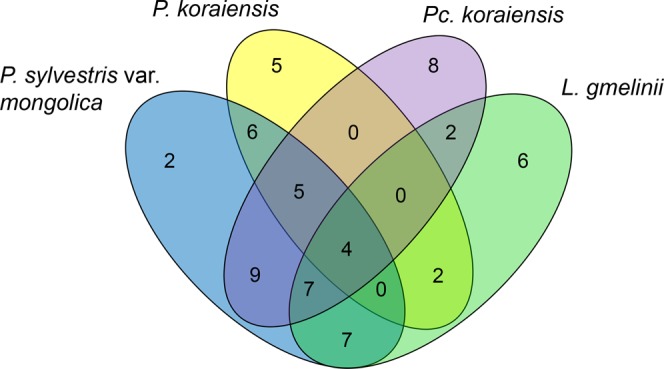
Distribution of endophytic fungal from four conifers in HG invaded by *S. noctilio*. Venn diagrams of the endophytic fungi were produced based on the endophytic fungal presence or absence data. The numbers correspond to number of species of endophytic fungi.

The diversity indexes of endophytic fungal communities isolated in the mixed coniferous forest invaded by *S. noctilio* in HG showed significant differences (Shannon’s diversity index: F = 5.77, p < 0.05; Evenness index: F = 94.20, p < 0.05; Richness index: F = 48.25, p < 0.05) (Table 2). Shannon’s index and Richness index of endophytic fungal were the highest in *Pc. koraiensis*, and those of *P. koraiensis* had the lowest value (Table 2). The similarity of the endophytic fungal communities of four tree species in HG was examined by NMDS using the Jaccard’s index (Fig. 4a) and the Bray–Curtis distance matrix (Fig. 4b). The results showed that the similarity value of endophytic fungal communities of four tree species was low (Fig. 4; Table S2 Supporting Information). Altogether, only four fungi species (*T. viride*, *T. atroviride*, *A. niger* and *F. tricinctum*) was shared by the four trees in HG (Fig. 5).

**Table 2:** The diversities of endophytic fungal communities from four conifers in HG.

Tree species	Shannon–Wiener index (*H*′)	Evenness index (*J*)	Richness index (*R*)
*Pc. koraiensis*	2.4989 ± 0.09^a^	0.8342 ± 0.011^b^	4.5135 ± 0.14^a^
*P. sylvestris* var. *mongolica*	2.2552 ± 0.13^ab^	0.8792 ± 0.004^a^	2.9430 ± 0.09^c^
*L. gmelinii*	2.0549 ± 0.04^b^	0.7253 ± 0.003^d^	3.5983 ± 0.05^b^
*P. koraiensis*	2.0414 ± 0.05^b^	0.7959 ± 0.005^c^	2.9229 ± 0.15^c^

Analysis of the similarity of endophytic fungal communities from four conifers in HG invaded by *S. noctilio*. The data were analyzed by one-way ANOVA followed by HSD test. The results are expressed as the mean ± SD (n = 9). The results followed by different letters are significantly different according to the HSD test (p < 0.05).

### The diversities and similarities of endophytic fungal communities from seven species of pine trees

The diversities of endophytic fungal communities from seven species of pine trees were significant difference (Shannon’s diversity index: F = 5.52, p < 0.05; Evenness index: F = 20.19, p < 0.05; Richness index: F = 21.51, p < 0.05) (Table 3). The diversity index value (2.2552 ± 0.13) of *P. sylvestris* var. *mongolica* was the highest in seven tree species; the three trees species in JDZ showed the lowest Richness index (*P. taeda*: 1.5647 ± 0.1; *P. elliottii*: 1.9557 ± 0.26; *P. massoniana*: 1.3854 ± 0.07) and Shannon’s diversity index (*P. taeda*: 1.8930 ± 0.08; *P. elliottii*: 1.8065 ± 0.07; *P. massoniana*:1.7163 ± 0.12). *Pinus taeda* had the highest Evenness index value (0.9103 ± 0.023). No significant differences in the three indices of endophytic fungi diversity were noted among *P. koraiensis*, *P. tabuliformis*, and *P. yunnanensis*. According to the result of NMDS, no clustering of the endophytic fungal communities from seven species of pine trees was apparent. (Fig. 4a,b). The endophyte communities from three trees in JDZ were similar. The similarity of endophyte communities from seven pine trees was more apparent from the Jaccard’s index coefficient than the Bray–Curtis (Fig. 4).

**Table 3:** The diversity indices of endophytic fungi from seven species of pine trees.

Tree species	Shannon–Wiener index (*H*′)	Evenness index (*J*)	Richness index (*R*)
*P. sylvestris* var. *mongolica*	2.2552 ± 0.13^a^	0.8792 ± 0.004^a^	2.9430 ± 0.09^a^
*P. koraiensis*	2.0414 ± 0.05^ab^	0.7959 ± 0.005^b^	2.9229 ± 0.15^a^
*P. tabuliformis*	1.9581 ± 0.04^ab^	0.8166 ± 0.004^b^	2.8358 ± 0.03^a^
*P. yunnanensis*	1.9808 ± 0.03^ab^	0.7971 ± 0.012^b^	2.6758 ± 0.20^a^
*P. taeda*	1.8930 ± 0.08^b^	0.9103 ± 0.023^a^	1.5647 ± 0.10^b^
*P. elliottii*	1.8065 ± 0.07^b^	0.7846 ± 0.006^b^	1.9557 ± 0.26^b^
*P. massoniana*	1.7163 ± 0.12^b^	0.8820 ± 0.009^a^	1.3854 ± 0.07^b^

Analysis of the similarity of endophytic fungal communities from seven species of pine trees. The data were analyzed by one-way ANOVA followed by HSD test. The results are expressed as the mean ± SD (n = 9). The results followed by different letters are significantly different according to the HSD test (p < 0.05).

## Discussion

Many studies have revealed that rich endophytic fungal communities have been isolated from a range of plants, which include beneficial species that have a negative impact on pests^29^. However, there is much less information on the relationship between endophyte communities and pest invasion. In this study, we analyzed the community structure and diversity of fungal endophytes from the host tree and other potential host trees of *S. noctilio* in China. We found that the different tree species did not share the most abundant and prevalent fungal species (Table 1). The number of endophytic fungal taxa species was higher in HG. However, the endophytes were most numerous in trees from JDZ. The analysis also revealed that the specificity of endophytic fungi for different coniferous trees varied (Table 3). These observations were similar to ones of a previous study in which the diversity and differences of endophytic fungal communities were significantly different among different tree species^30^. However, the endophytic fungal communities are different in different tree species, resulting in differences in the resistance of trees to pests^31^.

The diversity of endophytic fungal community in the host greatly affects the selection behavior of *S. noctilio* and the growth of its fungal symbiont^24,25^. *A. areolatum* is essential for egg eclosion (by creating a suitable environment) and larval nutrition, and contributes to increased adult insect size and reproductive success^31,32^. The female woodwasp probes the sapwood by shallow drilling into the host phloem using an ovipositor to find a suitable growth environment for the development of offspring and symbiotic fungus^33,34^. However, *A. areolatum* grows slowly and its ability to occupy an environmental niche is lower than that of other fungi. If various endophytic fungi colonize the host, interfering with the growth of the symbiont, the female will give up ovipositing and will only inject phytotoxic mucus, causing weakening of the host^35,36^. The CR and IR values of endophytic fungi of *P. sylvestris var. mongolica* was the lowest in mixed forests invaded by *S. noctilio* in HG. Among the pine species examined in the current study, the CR and IR were lower in *P. tabuliformis* and *P. sylvestris* var. *mongolica* than those of the other species (Fig. 1). Both of these tree species might constitute an advantageous environment for the adult female woodwasp to spawn and also benefit the growth of the woodwasp larvae.

On the other hand, no significant differences were observed in the CR or IR of *P. sylvestris* var. *mongolica* and the other trees species examined. Nevertheless, the primary endophytic fungal genera from the different tree species significantly differed. Many bioactive endophytes, including important biostimulants and bio-control agents, belong to such genera as *Trichoderma*, *Cordyceps*, *Metarhizium*, and *Beauvaria*, and nonpathogenic *Fusarium* species^37^. Therefore, endophytes belonging to these genera may present a strong resistance to the *S. noctilio* invasion. *Trichoderma* and *Fusarium* were the lowest in *P. tabuliformis*, *P. sylvestris* var. *mongolica* than in other tree species, while *Aspergillus* occupied a high percentage of *P. tabulaeformis*, *P. sylvestris* var. *mongolica*, and *P. yunnanensis* isolates (Fig. 2). *Trichoderma* exert a biological control effect against many pathogens and pests, and are characterized by rapid growth, antagonism, and parasitism^38,39^. *Trichoderma* were successfully applied for the treatment of pruning wounds on urban trees against colonization by wood decay fungi^40^. In fact, *Trichoderma* kill the mycelia of *A. areolatum*, while the inhibitory effect of *Aspergillus* against *A. areolatum* is less pronounced^25^. Therefore, *P. tabulaeformis*, *P. sylvestris* var. *mongolica* and *P. yunnanensis* might constitute more suitable hosts for the survival of the woodwasp larvae than other species. *L. procerum*, as an antagonistic fungus of *A. areolatum*, was isolated from *P. tabulaeformis* damaged by red turpentine beetle *Dendroctonus valens*^21,22,41^. But the fungi are rarely isolated from *P. tabulaeformis* in this study, and the reason may be due to the different health levels of *P. tabulaeformis* in the two experiments.

Ryan *et al*.^23^ reported that two species, *L. wingfieldii* and *O. minus*, can impact the selection of the site of woodwasp spawning. Volatiles of some blue stain fungi exert a repellent effect on the adult female woodwasp and, hence, influence the selection of the oviposition location^24,25^. In the current study, we found that endophytic fungi that can repel the woodwasp are relatively more rarely in *P. sylvestris* var. *mongolica* and *P. tabulaeformis* than in other trees (Tables 1; S1, Supporting Information). Interestingly, earlier host selection experiments similarly indicated that *S. noctilio* shows the highest preference for *P. tabulaeformis*, followed by *P. sylvestris* var. *mongolica*, and can complete its life cycle on *P. tabulaeformis* and *P. sylvestris* var. *mongolica*.

The species diversity of endophytic fungi isolated from the leaf is higher than that of those isolated from the stem, although the frequency of isolation is lower^42^. Endophyte diversity in the stem is higher than the diversity in the corresponding trunk^30^. The data presented in the current study suggested that the vertical distribution of endophytic fungi was influenced by the tree species (Fig. 3). The base segment of the tree trunk of *P. sylvestris* var. *mongolica* harbored an endophyte community that was more species-rich than those of the central and upper segments (submitted information). According to a field survey, the emergence holes of *S. noctilio* adults are mainly located in the upper segment of the *P. sylvestris* var. *mongolica* trunk, with more dead larvae infected by endophytic fungi in the base trunk than in the upper trunk. This might be because of a greater presence of endophytic fungi that inhibit the growth of *A. areolatum* in the base trunk than in the upper trunk. Except for *P. sylvestris* var. *mongolica* and *P. tabulaeformis*, the abundance of endophytic fungi in the upper segment was higher than in the middle and base segments of other tree species (Fig. S1, Supporting Information). This might be conducive to the invasion and colonization by *S. noctilio* of the upper trunk of *P. sylvestris* var. *mongolica* and *P. tabulaeformis*. Further, the different tree species did not share endophytes, illustrating that some endophytes exhibit a tree-specific or segment-specific preference, leading to, for example, the presence of endophytes in *P. sylvestris* var. *mongolica* that only weakly inhibit *S. noctilio*.

According to some studies, similar endophyte communities occur at close quarters^43–45^. In the current study, NMDS plots indicated that the diversity of the endophyte community was predominantly affected by tree species (different genera) in HG (Figs 4, 5). In addition, the endophytic fungal communities from seven species of pine trees has a large difference (Fig. 4). Concerning the distribution of *S. noctilio* in China, Carnegie^46^ and Ireland^18^ predicted that the regions from the northeast of Heilongjiang Province to the southwestern Yunnan Province (including the four areas evaluated in the current study) are climatically favorable for the establishment and persistence of *S. noctilio*, with all distribution records pointing to areas projected to be of moderate and high climatic suitability. Comparing the physical and chemical properties of host tree species in different areas invaded by *S. noctilio*, there is no obvious common characteristic^47,48^. *P. taeda* and *P. elliottii* are the host species of wasps in Canada and Brazil, and they are also distributed in the southern China. Therefore, it is necessary to study the effects of endophytic fungi in *P. taeda* and *P. elliottii* on the growth and development of *S. noctilio* larvae.

Although *S. noctilio* can endanger many pine trees worldwide, there are few systematic studies on the resistance of different hosts to *S. noctilio* and the growth of its symbiont^49^. The available research suggests that the resistance of the host tree itself likely contributes to maintaining the *S. noctilio* population in North America below damaging levels to a greater extent than the natural enemies of the woodwasp^20^. Because of the concealment of endophyte distribution in the host trunk tissue, their impact on natural communities and biodiversity may be easily overlooked. These inconspicuous mutualistic associations can, however, exert a tangible force on insect population dynamics that is qualitatively similar to that of natural enemies in maintaining the insect population in many ecosystems^50–53^. In the study, we analyzed the diversity of the communities of endophytic fungi from the established tree host and potential tree hosts of *S. noctilio*. From the perspective of endophytic fungi, preliminary analysis revealed that *P. tabulaeformis* enabled spawning of the adult female *S. noctilio* and survival of *S. noctilio* larvae. However, compared with other hosts of *S. noctilio*, *P. tabulaeformis* and *P. sylvestris* var. *mongolica* are characterized by the lowest wood hardness (as per Shore’s hardness method, below 20) and the thinnest phloem (below 6 mm), which are beneficial for the spawning of *S. noctilio*. Currently, *S. noctilio* is distributed in northeast China, the main distribution area of *P. sylvestris* var. *mongolica*, with gradual spreading to middle and southern China. The suitable hosts may encourage the spread of *S. noctilio* throughout China^46,48^. *Pinus tabulaeformis* is mainly distributed in the central regions and of China and, hence, it may be at a high risk of being attacked by *S. noctilio*.

## Materials and Methods

### Study sites and sample collection

**Figure 6:**
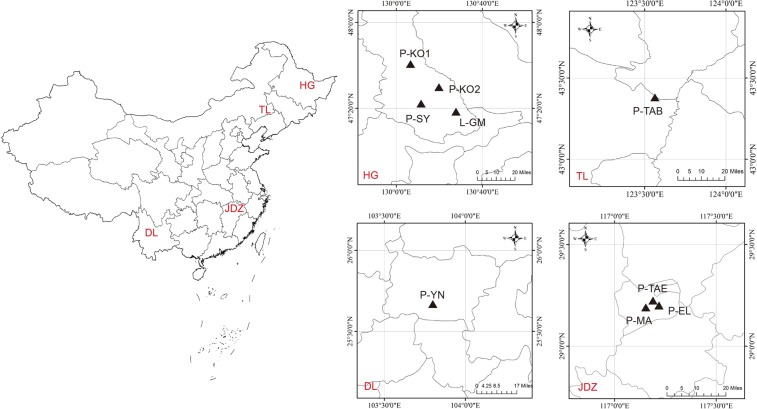
Map of the surveyed areas. Geographical locations of the nine coniferous tree species in four regions of China are shown. Sites: HG, Hegang; TL, Tongliao; DL, Dali; and JDZ, Jingdezhen. Tree species: P-SY, *P. sylvestris* var. *mongolica*; P-KO1, *P. koraiensis*; P-KO2, *Pc. koraiensis*; L-GM, *L. gmelinii*; P-TAB, *P. tabuliformis*; P-YN, *P. yunnanensis*; P-MA, *P. massoniana*; P-TAE, *P. taeda*; and P-EL, *P. elliottii*.

Material from nine coniferous species from four different climate zones in China was collected: from Hegang (HG: 47°12′11.4″N, 130°17′47.2″E): *P. sylvestris* var. *mongolica*, *P. koraiensis*, *Pc. koraiensis*, and *L. gmelinii*; from Tongliao (TL: 43°07′05.5″N, 123°28′34.8″E): *P. tabuliformis*; from Dali (DL: 25°36′21.8″N, 103°10′18.8″E): *P. yunnanensis*; and from Jingdezhen (JDZ: 29°16′01.0″N, 117°12′15.2″E): *P. massoniana*, *P. taeda*, and *P. elliottii* (Fig. 6). *S. noctilio* has invaded *P. sylvestris* var. *mongolica* in the mixed coniferous forest in HG. During the years 2015 and 2016, tree samples were collected from mixed forest (HG, TL, and JDZ) and pure forest stands (DL) (Fig. 6). Overall, 27 trees representing nine species (three repetitions per species) were randomly chosen from the four regions. Fresh wood samples were collected from three segments of the trunk, as follows: the base (0.1 m above ground), central segment (2.1 m above ground), and upper segment (4.2 m above ground). A trunk disk (10 cm-thick cross-section) was cut off from each segment. A bark layer more than 1 cm thick was removed from the disk using a sterile knife. Next, a 10 × 10 × 5 cm^3^ block was removed from each disk and sealed in a sterile vacuum bag. All samples were transferred to the laboratory of the Beijing Forestry University and stored at 4 °C until further analysis.

### Isolation and storage of the endophytic fungi

Endophytic fungi were isolated from the sample blocks using a surface sterilization method^54^. Each sample block was cut with a sterile pruner into 25 fragments (size: 5 mm^3^). Small fragments were surface-sterilized by dipping in a series of solutions (70% ethanol for 1 min, 12% sodium hypochlorite for 30 s, and 70% ethanol for 1 min). The pieces were then washed three times in sterile distilled water. Five surface-sterilized fragments were placed in a petri dish (90 mm), which contained potato dextrose agar (PDA: 200 g potato, 20 g glucose, 15 g agar, and 1 L distilled water) supplemented with 100 μg mL^−1^ ampicillin and 50 μg mL^−1^ chloramphenicol. All samples were incubated at 25 ± 1 °C and 70 ± 5% relative humidity (RH) for 1~4 weeks or until the emergence of the mycelia. Agar cubes (ca. 1 mm^2^) were removed aseptically from the edge of the colonies and transferred to fresh PDA plates. Each colony was transferred at least three more times until a visually uniform culture was obtained. For long-term preservation, the mycelia and spores were transferred to 20% glycerol in ultra-clean distilled water (v/v) and stored at −80 °C. Fungal cultures were generated on PDA slants in centrifuge tubes and stored under sterile mineral oil at 4 °C.

### Identification of endophytic fungi

The endophytic fungi were identified based on both morphology and internal transcribed spacer (ITS) sequencing. The endophytic fungi were first identified using ITS sequencing. DNA was extracted from fungal mycelia from fresh cultures, using the Extract-N-Amp tissue polymerase chain reaction (PCR) kit (Sigma–Aldrich Corporation, USA), following the manufacturer’s instructions. The fungal ribosomal ITS1 (ITS1), 5.8S (where present), and ITS2 regions were amplified using fungal-specific ITS1 and ITS4 primers^55^. The PCR reactions were carried out in a volume of 25 μL using 23 μL Golden Medal MIX (Thermo Scientific, USA), 1 μL of each primer, and 1 μL template DNA. Amplification was conducted using the following settings: an initial denaturation step of 98 °C for 2 min; followed by 30 cycles that included denaturation at 98 °C for 10 s, annealing at 50 °C for 15 s, and polymerization at 72 °C for 15 s; and a final extension step of 5 min at 72 °C.

The PCR amplification products were separated by electrophoresis on 1% (w/v) agarose gels and stained with ethidium bromide for visual examination. The PCR products were purified using the agarose gel DNA extraction kit (Takara, Japan) and sequenced at Qinke Biotech (Beijing, China). The sequences were submitted to BLAST search in the GenBank (http://blast.ncbi.nlm.nih.gov/Blast.cgi). Sequences sharing ≥99% similarity with a partial 28S rDNA sequence (ca. 600 bp) were considered as representing identical species.

When the sequences shared <99% similarity with known species, morphological features were used to identify the endophytic fungi. The following morphological features were evaluated: mycelium shape, mycelium surface texture, colony color, production of pigments and their diffusion in the medium, spore production, and mycelium growth rate on the PDA plates. The endophytic fungi that did not sporulate on this medium were transferred to the malt extract agar (MEA, 2%) plates and to plates with xylogen extracts of the host to activate sporulation. The following characteristics were evaluated for the anamorph: the conidiomata, conidiogenous cells, conidiophores, and conidia morphology (e.g. size, color, shape, and ornamentation). The following characteristics were evaluated for the teleomorph: the sporomata and their associated structures, and spore morphology^56^. Ultimately, the species of the endophytic fungal isolates were determined.

### Diversity analysis

The colonization rate (CR) was calculated as the number of fragments from which one or more endophytic fungi were isolated, divided by the total number of incubated fragments^57^. The isolation rate (IR) was defined as the number of endophytic fungi isolated, divided by the total number of fragments incubated^58,59^. The CR and IR were analyzed using one-way ANOVA. The differences between mean values were evaluated using Tukey’s honestly significant differences (HSD) test. A Chi-square test was applied to analyze the data for some tree species. The statistical analyses were performed using the IBM SPSS Statistics version 23.0 (Chicago, IL, USA). The differences and distribution of endophytic fungi isolated from each tree species were examined using the range diversity analysis^60^. The analysis of variance was used to test for differences in endophyte richness among tree species.

The diversity of endophytic fungi isolated from seven species of pine trees and four conifers in HG were evaluated using the Shannon–Weiner Index (*H′*), Evenness Index (*J*), and Margalef richness index (*R*); the differences between the indices were analyzed using one-way ANOVA.

$$H^{\prime} =-\,{\rm{\Sigma }}\,(Pi\times \,\mathrm{ln}\,Pi)$$

$$J=H^{\prime} \,/\mathrm{ln}(S)$$

$$R=(S-1)/\mathrm{ln}\,N$$

$$Pi=Ni/N$$

where *N* is the total number of individuals; *Ni* refers to the number of individuals; and *S* indicates the total number of species.

The differences in endophyte community structure identified at different trunk heights of each tree species and the four conifers in HG were analyzed respectively using Venn diagrams (GraphPad Prism 7, San Diego, CA, USA). Endophyte communities were compared by using non-metric multidimensional scaling (NMDS), using the R package VEGAN (version 2.3-0). Two NMDS plots were constructed, each based on a different calculated similarity index: the Jaccard’s index, based on the presence/absence of taxa among tree species^61^; and the Bray–Curtis coefficient, based on the incidence and abundance of taxa in the tree species^61^.

### Informed consent

All experimental protocols were approved by Key Laboratory for Silviculture and Conservation of Ministry of Education, Beijing Forestry University, Beijing, China.

All the methods were carried out in accordance with the relevant guidelines and regulations.

## Supplementary information

Supplementary

## Data Availability

We declare that all the date in this study were available.
