# Supplementary material for: Effects of endophytic fungi diversity in different coniferous species on the colonization of Sirex noctilio (Hymenoptera: Siricidae)
Source: Sci Rep. 2019 Mar 25;9:5077. doi: 10.1038/s41598-019-41419-3 (PMC6433867; doi:10.1038/s41598-019-41419-3)
Supplement: Supplementary file 1 — Supplementary [file 41598_2019_41419_MOESM1_ESM.docx]

**Effects of endophytic fungi diversity in** **different coniferous species on the colonization of** ***Sirex noctilio*(Hymenoptera: Siricidae)**

**Lixiang Wang****^1^, Lili Ren^1,2^,** **Chunchun Li^3^, Chenglong Gao^1^, Xiaobo Liu^1^, Wang Ming^1^ and Youqing Luo^1,2^**

1 Beijing Key Laboratory for Forest Pest Control, Beijing Forestry University, Beijing 100083, China.

2 Sino-France Joint Laboratory for Invasive Forest Pest in Eurasia, Beijing 100083, China.

3 Shangluo Agricultural Mechanical Technology Extension Station, Shangluo 726000, China.

**Table S1.** Diversity of fungal endophytic communities of nine trees species

| **Tree species** | **No. of isolates** | **No. of genus** | **No. of species** | **No. of unique** | **dominating fungi** |
| --- | --- | --- | --- | --- | --- |
| *P. tabuliformis* | 82 | 8 | 11 | 6 | *Aspergillus vensicolon*  *Aspergillus jensenii* |
| *P. sylvestris* var. *mongolica* | 137 | 9 | 13 | 2 | *Aspergillus niger*  *Chaetomium globosum*  *Sphaeropsis sapinea* |
| *P. koraiensis* | 153 | 7 | 13 | 4 | *Trichoderma atroviride*  *Trichoderma viride*  *Ophiostoma minus* |
| *P. yunnanensis* | 169 | 10 | 13 | 4 | *Penicillium chrysogenum*  *Aspergillus niger* |
| *P. massoniana* | 188 | 6 | 9 | 1 | *Trichoderma citrinoviride*  *Penicillium crustosum*  *Sphaeropsis sapinea* |
| *Pc. koraiensis* | 197 | 15 | 20 | 8 | *Trichoderma atroviride*  *Fusarium tricinctum*  *Sphaeropsis sapinea*  *Paraconiothyrium* sp. |
| *L. gmelinii* | 218 | 9 | 17 | 6 | *Mucor plumbeu*  *Fusarium tricinctum*  *Cytospora chrysosperma*  *Trichoderma atroviride* |
| *P. taeda* | 224 | 5 | 10 | 3 | *Trichoderma harzianum Lasiodiplodia theobromae*  *Trichoderma viride* |
| *P. elliottii* | 258 | 6 | 11 | 2 | *Trichoderma citrinoviride*  *penicillium rolfsii*  *Aspergillus niger* |

**Table S2** Similarity coefficients of endophytic fungi from four conifer species in HG

| Tree species | *P. sylvestris* var. *mongolica* | | *P. koraiensis* | *L. kaempferi* | *P. sperata* |
| --- | --- | --- | --- | --- | --- |
| *P. sylvestris* var. *mongolica* | | 1.0000 | − | − | − |
| *Pinus koraiensis* | 0.3000 | | 1.0000 | − | − |
| *Larix kaempferi* | 0.3043 | | 0.2500 | 1.0000 | − |
| *Picea asperata* | 0.4167 | | 0.1333 | 0.4737 | 1.0000 |

**Figure S1**

**Figure S1.** Isolation rates of endophytes according to different trunk heights (Base, Central, and Upper) of the different tree species.
